# High muscular fitness level may positively affect bone strength and body composition in children with overweight and obesity

**DOI:** 10.1007/s11657-024-01405-3

**Published:** 2024-06-10

**Authors:** Cristina Comeras-Chueca, Lorena Villalba-Heredia, Gabriel Lozano-Berges, Ángel Matute-Llorente, Jorge Marín-Puyalto, Germán Vicente-Rodríguez, José A. Casajús, Alejandro González-Agüero

**Affiliations:** 1 EXER-GENUD “Growth, Exercise, NUtrition and Development” Research Group, Universidad de Zaragoza, C/ Pedro Cerbuna Nº 12, 50009 Saragossa, Spain; 2 EXERNET Red de Investigación en Ejercicio Físico y Salud, Saragossa, Spain; 3 Faculty of Health Science, Faculty of Medicine, Universidad de Zaragoza, Saragossa, Spain; 4 Department of Physiatry and Nursing, Faculty of Health and Sport Science (FCSD), Universidad de Zaragoza, Saragossa, Spain; 5 Instituto Agroalimentario de Aragón-IA2 (Universidad de Zaragoza-CITA), Saragossa, Spain; 6 Centro de Investigación Biomédica en Red de Fisiopatología de La Obesidad y Nutrición (CIBERObn), Madrid, Spain

**Keywords:** Childhood obesity, Body composition, Bone health, Muscular fitness, Youth

## Abstract

**Summary:**

Muscular fitness plays a major role in bone health and body composition in overweight and obese children. It is key that the development of this muscle fitness is affected by absolute isometric strength and dynamic strength.

**Purpose:**

To compare bone health and body composition between overweight/obese children considering muscular fitness (MF) levels, and to investigate whether weight-bearing dynamic or absolute isometric strength, both involved in the development of this muscular fitness, are more related with bone health.

**Methods:**

MF of 59 overweight or obese children (10.1 ± 0.9 years, 27 females) was measured by a countermovement jump (CMJ), handgrip, and maximal isometric strength of knee extension. Participants were divided into four groups depending on their MF level performing a cluster analysis: 16 children with high MF (HMF) in all tests, 18 with high performance in isometric strength (HIS), 15 with high performance in CMJ (HCMJ) and 10 low isometric and low dynamic force values (LMF). Body composition values were measured by dual energy X-ray absorptiometry, and bone strength values were assessed by peripheral quantitative computed tomography. Motor skills were evaluated using TGMD-3. Multivariate analysis of covariance test was applied to analyse bone strength differences between children in the different MF groups, using maturity offset, height and weight as covariates, and correlations were investigated.

**Results:**

HMF excelled in bone health. HIS had higher cortical bone area, periosteal circumference, bone mass, polar strength strain index and fracture load than LMF, while HCMJ only showed better results in trabecular bone area than LMF. HMF had significantly better values of fracture load and periosteal and endosteal circumferences than HCMJ, but not than HIS.

**Conclusions:**

High MF level shows positive effects on bone health in overweight/obese children. Those with highest isometric strength had better bone health compared to those with higher dynamic strength.

**Trial registration:**

The research project was registered in a public database Clinicaltrials.gov in June 2020 with the identification number NCT04418713.

## Introduction

Obesity has become a major global health challenge because of its increased prevalence [1]. Although bone mineral content (BMC) is higher in obese children than in normal-weight peers because body adiposity represents a mechanical load [2], bone fractures of the lower limb are much more frequent in obese children. This is due to the excessive mechanical loading caused by excessive body mass and hormonal negative effects such as an increase in proinflammatory cytokines, leptin and adipokines [3, 4]. A longitudinal study in overweight children showed that stress–strain index (SSI), a measure indicating the mechanical strength and resistance of the bone to deformation under various loading conditions, was low when adjusted for the body fat in children with excess weight, suggesting that bone strength may be not adapted to body fat excess [5]. Likewise, children with higher body adipose tissue show a reduced volumetric bone mineral density (vBMD), geometry and indices of bone strength, suggesting a mismatch between gains in body mass and acquisition of vBMD and geometry during growth [5].

Children and adolescents with overweight or obesity exhibit higher absolute maximum muscular strength. However, when normalized to body mass or lean mass, they appear weaker, likely due to factors such as reduced mobility, neural adaptations, and changes in muscle morphology [6, 7]. Consequently, their performance on strength tests involving body weight movement, such as jumping, is impaired [8]. Conversely, they show improved performance on strength tests that don’t involve lifting their body, such as isometric knee extension strength or handgrip tests [8]. Likewise, overweight and obesity are associated with deficient muscular strength and endurance [9, 10]. In addition, these children display poorer muscle tissue composition with higher intramuscular fat infiltrations, lower muscle power and altered motor unit recruitment, leading to impaired muscle activation [11]. Thus, children and adolescents with overweight or obesity are more likely to experience the paediatric dynapenia condition of the paediatric inactivity triad (PIT) [12]. Sarcopenia can be defined as age-related muscle loss; however, dynapenia is the common muscle loss in adolescence as due to physical inactivity among other factors [12].

Higher muscular fitness (MF) may attenuate the adverse hormonal effect of childhood obesity on bone mass [13]. There are several tests to measure MF, as dynamic vs. isometric, or moving the body weight vs. with external load or as muscle activity against resistance without movement of the body part. MF has been related to a higher areal bone mineral density (aBMD) later in life [14] and better bone health during growth [15, 16]. Evidence has shown that weight-bearing loading in resistance training is the best strength type for the development and production of bone tissue [14].

The aim of this study was to compare bone health and body composition between overweight and obese children with different MF levels, also considering covariates such as maturity offset, height and weight. Another important aim of this article was to investigate which type of strength between weight-bearing dynamic force and absolute isometric force is more closely related to bone health in children with overweight or obesity.

## Methods

### Participants

A total of 59 children with overweight or obesity were included in this study. MF was measured by a countermovement jump (CMJ), handgrip and maximal isometric strength of knee extension. After performing a cluster analysis, participants were split into four groups depending on their MF level as follow: 16 children with high MF (HMF) in both isometric (knee and handgrip) and CMJ (10.7 ± 0.6 years, 8 males), 18 with high performance in isometric strength (HIS) (10.2 ± 0.9 years, 10 males), 15 with high performance in CMJ (HCMJ) (9.7 ± 0.9 years, 10 males) and 10 with low MF (LMF) (9.5 ± 0.6 years, 4 males).

In addition, a post hoc power analysis was conducted using G*Power software to evaluate the sample size. The analysis focused on the fracture load, a critical variable in this research. With a partial eta squared of 0.604, significance level (*α*) at 0.05 and a total sample size of 58 across four groups, the analysis resulted in a non-centrality parameter of 21.129 and a critical *F* value of 2.02. The achieved power was 0.844, indicating a high likelihood of detecting the anticipated effect.

This study was performed in accordance with the ethical guidelines for human research outlined by the Declaration of Helsinki (revision of Fortaleza 2013) [17] and the Declaration of Taipei [18] and was reviewed and approved by the Research Ethics Committee of the Government of Aragon (certificate nº 11/2018, CEICA, Spain). All participants and their parents or legal custodians were informed of the nature and possible risks of the measures before their written informed consent was obtained. The research project was registered in a public database Clinicaltrials.gov (identification number NCT04418713). The Strengthening the Reporting of Observational Studies in Epidemiology (STROBE) Statement was used as a guideline for reporting observational data [19].

### Inclusion criteria

Participants had to be between 9 and 12 years, in Tanner stage I or II and not having had menarche, and with overweight or obesity established following the cut points of Cole et al. [20]. Tanner’s stage was evaluated by a medical doctor by direct observation. Volunteers suffering from pathologies that worsen with exercise or having any other contraindications for its practice were excluded from the present study. In addition, children could not be included if they were participating in regular high-level or high-intensity extracurricular PA, following any special dietary regime, and taking any medication that might interfere with the evaluated variables.

### Anthropometric measurements

Height (stadiometer SECA 225, SECA, Hamburg, Germany) was measured without shoes and the minimum clothes to the nearest 0.1 cm and weight to the nearest 0.1 kg (SECA 861, SECA, Hamburg, Germany). Body mass index (BMI) was calculated as weight (kg) divided by squared height (m^2^).

### Muscular fitness (MF)

#### Countermovement jump (CMJ)

CMJ (cm) was evaluated using a Kistler force plate 9260AA (Kistler Holding AG, Winterthur, Switzerland) to measure the vertical jump height in centimetres. Children were in a standing position with both hands on their hips to isolate the lower limb action. The lower extremities were positioned parallel to each other, and the feet were flat on the force plate. For the performance of the countermovement, children were asked to go down fast and not to stop after going down. This dynamic motion, known as the countermovement, is characterized by a continuous and fluid transition from the descending to the ascending phase. As depicted in the Image 1, during the flight phase, a triple extension (ankle, knee, and hip) is required, and the hands should be kept on the hips. Three attempts were permitted, and the best performance was recorded for further analysis. Normalized scores were obtained based on the results of the study performed by Focke et al. [21], adjusting the value to the subject's body mass. The jump height was calculated from the flight time using the following Eq. [22]:

$${h}_{t}=\frac{g{({t}_{v})}^{2}}{8}$$

where ${h}_{t}$ is the jump height, *g* is the acceleration due to gravity (approximately 9.8 m/s^2^ on the surface of the Earth) and ${{t}_{v}}^{2}$ Flight time (in seconds) squared.

**Figure:**
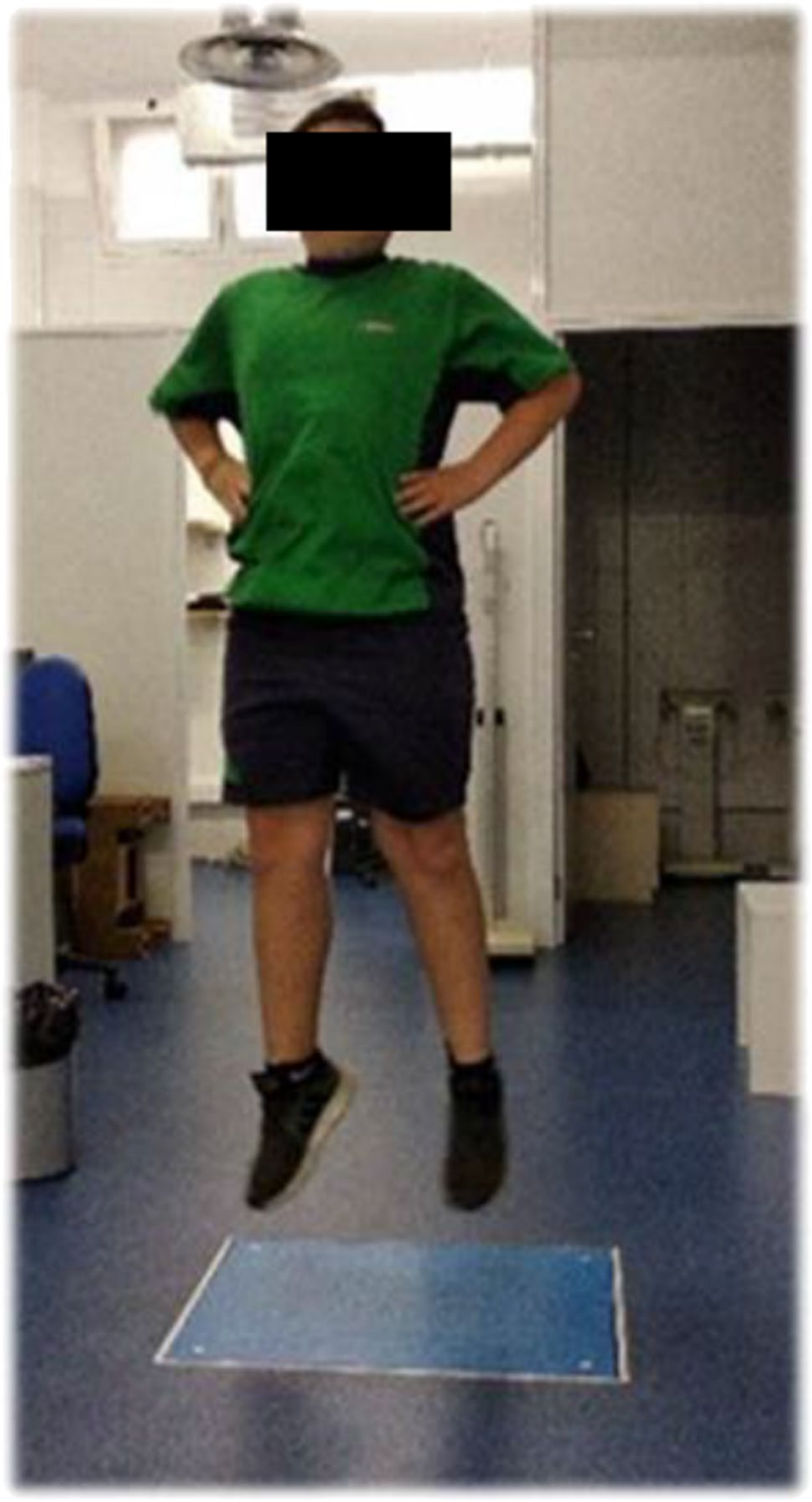
Participant performing a CMJ jump test

#### Maximal isometric strength of knee extension

Maximal isometric strength of knee extension (kg) was measured by a signal-frame gauge (Universidad de Zaragoza, Zaragoza, Spain). Children started from a sitting position with their knees flexed 90°. An anchorage was placed on the anterior distal third of the tibia. This anchorage was connected to the strain gauge, registering force data during the time that the participant had to perform the maximum knee extension until the force curve began to decrease. Three attempts were permitted for each leg, with the best performance recorded from each leg. The mean of the best attempt for each leg was obtained to quantify isometric knee extension strength. Normalized scores were obtained based on the reference values of the study performed by Beenakker et al. [23], adjusting the value to the subject's body mass.

#### Handgrip strength

Handgrip strength test (kg) was measured using a handgrip dynamometer (TKK 5001, grip A; Takei). Children were in a standing position maintaining the arm of the tested side straight down with the shoulder slightly abducted (~ 10° not touching the rest of the body), the elbow in 0° of flexion, the forearm in neutral position and the wrist in 0° of flexion. The best value of three attempts with each hand was used. Handgrip strength percentiles by age and sex were calculated based on normative values of children and adolescents aged 9–17 years representing 24 countries proposed by Tomkinson et al. [24].

### Bone assessment by pQCT and DXA

Bone strength indexes, bone morphometry, bone area, strength indexes and trabecular bone microarchitecture were measured at non-dominant tibia using a Stratec XCT-2000 L Peripheral quantitative computed tomography (pQCT) scanner (Stratec Medizintechnik, Pforzheim, Germany). The device is a translate-rotate, small bore computed tomography scanner that obtains a trans-axial image. The pQCT was calibrated daily based on a quality control phantom provided by the manufacturer (Stratec Medizintechnik, Pforzheim, Germany). Coefficients of variation for each pQCT variable in our laboratory for each variable have been already reported [25, 26] and ranged between 0.82 and 2.38% for bone variables and between 1.69 and 3.88% for muscle area and fat area, respectively.

The non-dominant leg was the contralateral leg to the one that would be used for kicking a ball and was determined by asking which leg would be used to kick a ball. Participants were seated on a chair adjustable to the body proportions of each participant. Tibia length was measured from the medial knee joint cleft to the medial malleolus of the tibia, and it was always performed by the same technician using a wooden ruler. The assessed leg was centrally placed in the imaging field, and the foot and knee were secured to minimize movement. The scanner was positioned on the distal end of the tibia, and a coronal computed radiography (scout view) was performed to manually locate the reference line on the midpoint of the distal tibia endplate.

The bone measurements were performed at 8%, 38% and 66% of the tibia length. Following the 2013 ISCD Pediatric Official Positions [27], the assessed variables at the 8% site were total bone area, trabecular area, total vBMD and trabecular BMD. The measured variables at the 38% were total bone area, total vBMD, cortical thickness (CRT_THK38), periosteal circumference (PERI38), endosteal circumference (ENDO38), cortical bone area, cortical bone density and polar strength strain index (SSIPOL38). At 66% site of the tibia, muscular and subcutaneous and intramuscular adipose tissue and total bone area were obtained. Images were analysed with version 6.20 of the manufacturer’s software.

Dual energy X-ray absorptiometry (DXA) scans were performed with the paediatric version of the QDR-Explorer software (Hologic Corp., Software version 12.6.1, Bedford, MA, USA) for the whole body (and its sub-regions). Total body less head (TBLH), legs (calculated as a mean of both legs) and arms (calculated as a mean of both arms) lean mass were obtained from whole body scans for BMC and aBMD. All DXA analyses were performed by the same trained researcher. The coefficient of variation of DXA in the laboratory for bone area was 2.6%, for BMC was 2.3% and for aBMD was 1.3% [28]. Furthermore, fat and lean mass index normalized to height was calculated, and normalized scores were obtained based on the reference values of the study performed by Weber et al. [29]. A report of the DXA analysis was given to each participant.

### Motor skills

Motor skills were assessed in 32 participants, using the Test for Gross Motor Development-3rd Edition (TGMD-3), which measures 13 fundamental skills, subdivided into locomotor and object control domains [30]. Researchers who evaluated the motor competence with this test completed the four reliability videos evaluation to check the consistency of administration and coding and the intra- and inter-observer variability. The score of the tests, result coding and percentiles were obtained based on the examiner’s manual [31].

### Statistical analyses

The Statistical Package for the Social Sciences (SPSS) version 23.0 (SPSS Inc., Chicago, IL, USA) was used to perform all the statistical analyses. Statistical significance was set at *p* < 0.05 in all tests. Data are presented as mean ± standard deviation (SD). All variables showed normal distribution by the Kolmogorov–Smirnov test. Differences between groups for descriptive characteristics were investigated.

Cluster analysis was performed to identify groups of physical fitness and compare bone variables among groups. As no sex-based differences for bone variables were found, cluster analyses were performed for the entire sample. Cluster analysis was performed to divide the participants into groups depending on their MF level according to the performance in handgrip, maximal isometric strength of knee extension and CMJ test. The methodology of clustering employed in the studies by Prokasky et al. [32] and Sanson et al. [33] was followed, and hierarchical clustering and *K*-means clustering were performed. Outliers for handgrip and CMJ test were examined to reduce the sensitivity of the Ward’s method to outliers, and no outliers were found. Firstly, hierarchical cluster analysis was performed to determine the number of clusters. Four groups were showed by the dendrogram obtained from this analysis. Afterwards, *K*-means clustering analysis was run using as non-random starting points the cluster centres obtained by the previous Ward’s hierarchical procedure. Two subsamples were randomly obtained from the whole sample of this study, and cluster analysis was repeated to check the stability of these clusters. Subsequently, Cohen’s Kappa coefficient (κ) was used to measure the agreement between the original cluster and the two clusters obtained by subsamples, showing a perfect agreement as the result was 1.

Therefore, four groups were obtained, and multivariate analysis of covariance (ANCOVAs) test was applied to analyse bone strength differences between overweight or obese children who had different MF, using maturity offset, height and weight as covariates. Effect size statistics were reported as partial eta squared (η^2^_p_) for ANCOVAs, and according to the cut-off established by Cohen et al. [34], the effect size d can be small (0.01–0.06), medium (0.06–0.14) or large (> 0.14).

Correlations were examinated between fat, lean and bone results and dynamic and isometric strength together with motor skills.

## Results

**Fig. 1:**
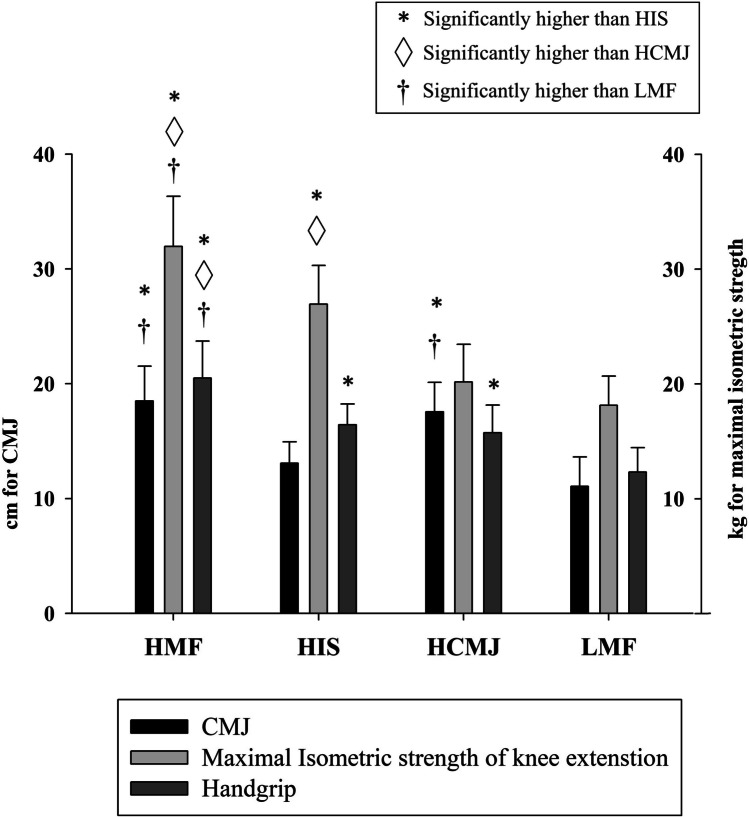
Descriptive muscular fitness data of the four groups after clustering by muscular fitness. CMJ, countermovement jump; HCMJ, high score in countermovement jump; HIS, high score in isometric strength; HMF, high score in all strength tests; LMF, low score in all strength tests

Descriptive characteristics of the whole sample and of the sample clustered by MF are presented in Table 1. A total of 59 children with overweight or obesity were included in the study with a baseline mean age of 10.1 ± 0.9 years (from 9 to 12). The four groups created after clustering can be seen differentiated by performance in the different muscle fitness tests in Fig. 1.

**Table 1:** Summary of descriptive characteristics of the participants

	All participants (*N* = 59)	HMF (*N* = 16)	HIS (*N* = 18)	HCMJ (*N* = 15)	LMF (*N* = 10)
Age (years)	10.1 ± 0.9	10.7 ± 0.6	10.2 ± 0.9	9.7 ± 0.9	9.5 ± 0.6
Weight (kg)	54.5 ± 9.9	60.2 ± 9.1	55.8 ± 7.4	49.6 ± 9.2	50.6 ± 12.0
Height (cm)	145.1 ± 7.5	149.4 ± 6.5	144.4 ± 5.6	143.9 ± 8.8	141.1 ± 8.0
BMI (kg/m^2^)	25.7 ± 3.0	26.8 ± 2.8	26.8 ± 2.7	23.7 ± 2.1	25.1 ± 3.4
Fat percentage (%)	40.8 ± 3.9	39.3 ± 3.3	42.4 ± 2.7	38.6 ± 3.6	43.7 ± 4.2
Waist circumference (cm)	79.1 ± 7.3	81.7 ± 7.1	80.8 ± 6.0	75.7 ± 6.1	77.1 ± 9.5
Hip circumference (cm)	91.0 ± 7.2	93.9 ± 6.1	93.0 ± 5.6	86.8 ± 7.0	88.9 ± 9.0

*BMI* body mass index, *HCMJ* high score in countermovement jump, *HIS* high score in isometric strength, *HMF* high score in all strength tests, *LMF* low score in all strength tests

### Relationship between groups after clustering by muscular fitness and adipose tissue

**Fig. 2:**
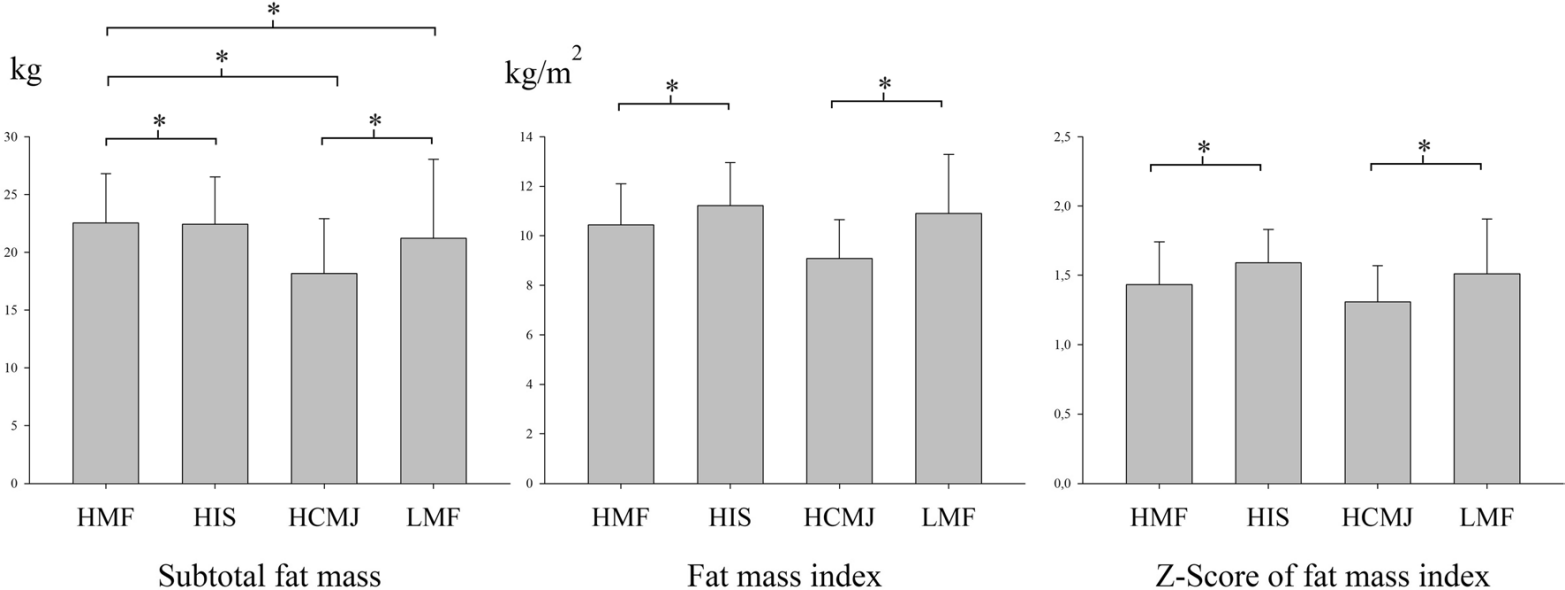
Relationship between muscular fitness and body fat. HCMJ, high score in countermovement jump; HIS, high score in isometric strength; HMF, high score in all strength tests; LMF, low score in all strength tests. *Significant differences between groups (*p* < 0.05)

Comparing body fat between groups, HMF group had higher body fat compared to the other groups (HIS, HCMJ and LMF), and LMF had higher body fat that HCMJ (η^2^p = 0.302). However, regarding to adipose tissue index or *z*-score of adipose tissue index, HIS had higher values compared to HMF group, and HCMJ showed lower fat than LMF (η^2^p = 0.347 and η^2^p = 0.221, respectively). These results are shown in Fig. 2.

### Comparison between groups after clustering by muscular fitness and lean mass

**Fig. 3:**
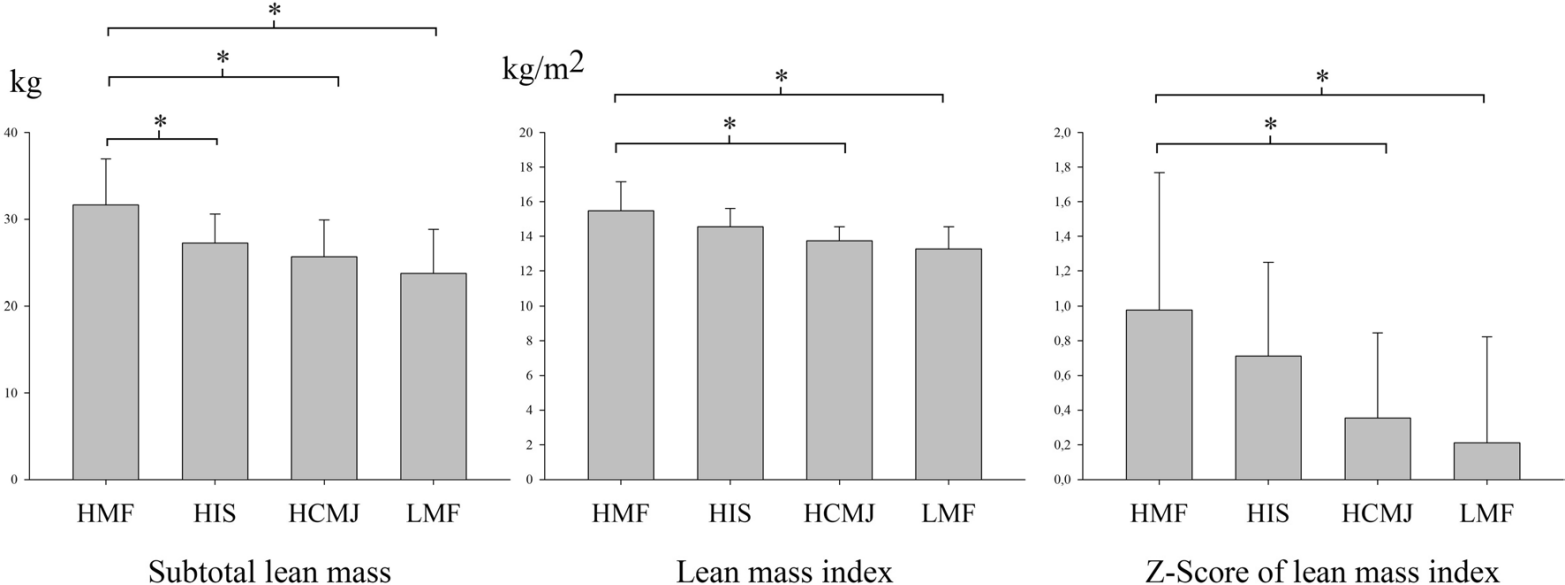
Relationship between muscular fitness and lean fat. HCMJ, high score in countermovement jump; HIS, high score in isometric strength; HMF, high score in all strength tests; LMF, low score in all strength tests

As shown in Fig. 3, when investigating lean mass results between groups according to MF, the results were similar to those obtained by comparing adipose tissue. HMF group had higher lean mass compared to the other groups (HIS, HCMJ and LMF) (η^2^p = 0.395). Nevertheless, regarding to lean mass index or *z*-score of lean mass index, there were no significant differences between HMF and HIS, while HMF had significantly higher lean mass index and *z*-score of lean mass index than HCMJ and LMF (η^2^p = 0.416 and η^2^p = 0.186, respectively).

### Relationship between groups after clustering by muscular fitness and bone health

**Fig. 4:**
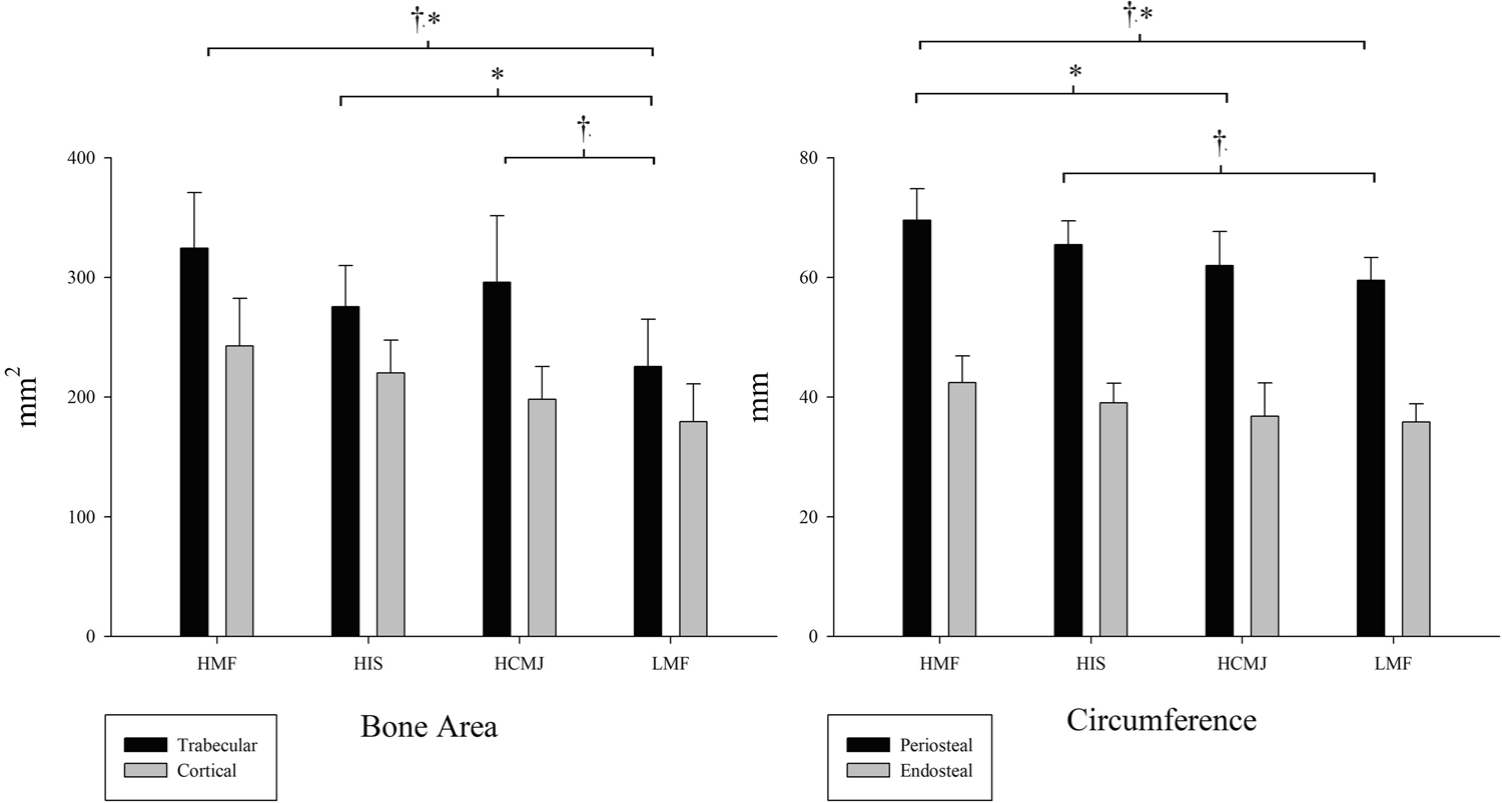
Relationship between muscular fitness and bone microarchitecture. HCMJ, high score in countermovement jump; HIS, high score in isometric strength; HMF, high score in all strength tests; LMF, low score in all strength tests. †Significant differences in trabecular bone area and periosteal circumference. *Significant differences in cortical bone area and endosteal circumference

Results related to bone health and the microarchitecture can be seen in Fig. 4. Looking inside the bone, specifically to the cortical and trabecular bone area, HMF and HIS groups showed higher values of cortical bone area than LMF (η^2^p = 0.224), but there were no significant differences between HCMJ and LMF. Instead, HMF and HCMJ groups had better results for trabecular bone area compared to LMF (η^2^p = 0.339), but there were no significant differences between HIS and LMF.

The periosteal and endosteal circumference was higher for HMF group than for HCMJ and LMF groups (η^2^p = 0.295 and η^2^p = 0.173, respectively) but not than HIS. As well, a higher periosteal circumference was shown for HIS compared to LMF.

**Fig. 5:**
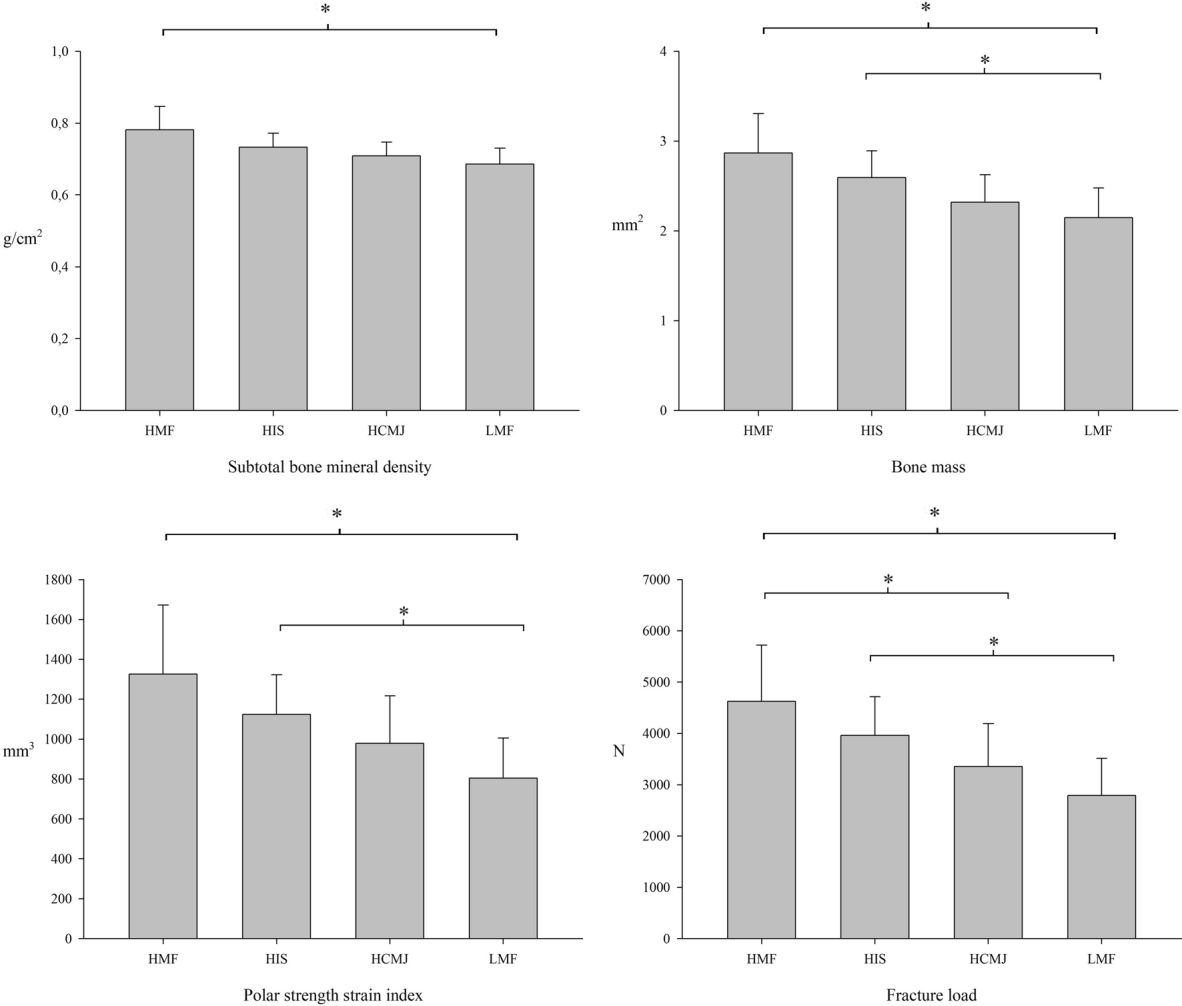
Relationship between muscular fitness and bone health. *HCMJ*, high score in countermovement jump; *HIS*, high score in isometric strength; HMF, high score in all strength tests; LMF, low score in all strength tests

Some outcomes closely related to bone health are aBMD and bone mass, and the results are shown in Fig. 5. Comparison between MF groups found that HMF for aBMD and HMF and HIS for bone mass showed higher values then LMF.

On the other hand, some of the bone outcomes most closely related to bone health were bone strength variables, resistance to bending and torsion of the bone (polar strength strain index) and force that must be applied to the bone to break the bone (fracture load). HMF and HIS showed higher values and, therefore, stronger bones compared to LMF for both polar strength strain index and fracture load (η^2^p = 0.205 and η^2^p = 0.197, respectively). In addition, HMF had higher fracture load than HCMJ.

### Correlations between muscular fitness, bone health and body composition

Correlations were performed to research the relationships between body composition, MF, motor skills and bone health. The correlations were conducted using the data from the entire sample, rather than the subgroups derived from the cluster analysis. Positive relationships were found between fat or lean mass and bone results (*r* ranged from 0.27 to 0.72 for adipose tissue and from 0.32 to 0.81 for lean mass, *p* < 0.05), specifically with aBMD, periosteal and endosteal circumferences, trabecular and cortical bone area, cortical thickness, polar strength strain index and fracture load, in addition to cortical thickness for lean mass (*r* = 0.20, *p* < 0.05). Adipose tissue was inversely correlated CMJ and motor skills (*r* =  − 0.41 and* r* =  − 0.55 respectively, *p* < 0.05).

Direct correlations between dynamic strength measured by CMJ with aBMD, trabecular bone area, cortical thickness and motor skills (*r* ranged from 0.32 to 0.63, *p* < 0.05) and inverse correlations with TBLH fat, adipose tissue index and *z*-score of adipose tissue index were found (*r* ranged from − 0.43 to − 0.55, *p* < 0.05). Isometric strength measured by handgrip and maximal isometric strength of knee extension was positively correlated with aBMD, periosteal and endosteal circumference, trabecular and cortical bone area, cortical thickness, polar strength strain index and fracture load, TBLH lean mass, lean mass index and *z*-score of lean mass index (*r* ranged from 0.32 to 0.72, *p* < 0.05).

Lastly motor skills had an inversely relation with TBLH fat, adipose tissue index and *z*-score of adipose tissue index (*r* ranged from 0.41 to 0.49,* p* < 0.05) and directly with CMJ (*r* = 0.63, *p* < 0.001).

## Discussion

The main finding of the present study is that higher MF can be related to improved bone health, considering maturity offset, height and weight as confounding variables. These results are observed with HMF showing better results than LMF for cortical and trabecular bone area, periosteal and endosteal circumferences, aBMD, bone mass, polar strength strain index and fracture load.

Another important aim of this article was to examine which type of strength between weight-bearing dynamic force or absolute isometric force is more closely related to bone health in children with overweight or obesity. The results obtained seem to indicate that absolute isometric strength tends to be more strongly related to bone health. This can be seen in the results which show that HIS had higher values of cortical bone area than LMF, although HCMJ also showed better results in trabecular bone area than LMF. However, this tendency for the HIS group to have an advantage over HCMJ is also shown in other bone variables in which HIS showed higher periosteal circumference, bone mass, polar strength strain index and fracture load than LMF, while there was no significant difference between HCMJ and LMF. In addition, HMF had significantly better values of fracture load and periosteal and endosteal circumferences than HCMJ, but not than HIS, which evidence a better bone health of HIS compared to HCMJ.

The results of the present study were in the same direction as previous scientific literature [13], which showed a strong relationship between MF and bone health, being able to identify muscle strength as a skeletal health marker during development not only because the mechanical stimulus but also because the physiological effects. However, this is the first time to be analysed in a population of children with overweight or obesity.

A meta-analysis showed significant correlation with a moderate-large effect size between MF and aBMD (*r* = 0.166; 95% CI 0.086 to 0.243) at follow-up [14]. There is also scientific evidence indicating that weight-bearing strengthening enhances bone mass and bone structure during childhood [35]. However, according to the results of the present study, weight-bearing strength is less related to bone health in children with overweight or obesity, probably due to a lack of osteogenic stimulation because of low motor skills, as supported by the inverse relationship found between motor skill and the variables body fat, body mass index and *z*-score of body mass index and by the direct relationship between motor skill and CMJ performance.

MF demonstrated as well a high discriminatory ability for bone health and, among the tests to determine MF, handgrip showed the highest discriminatory ability [36]. Children with overweight and obesity stand out for their absolute non-weight-bearing strength, showing high values of handgrip in general and hence a lower risk of poorer bone development. Therefore, when classifying with the cut-off points created by Saint-Maurice et al. [37] to stratify the risk of proper bone development in childhood, children who are overweight or obese are more likely to be categorized as low risk.

In this regard, another current systematic review with meta‑analysis concludes that muscular strength should be considered as a useful skeletal health marker during development and this strengthening is not only a pure mechanical stimulus, but also involve muscle glycogen metabolism and systemic-related changes [16]. That means that not only the weight-bearing strength exercises enhance bone health, so having a high isometric strength can be related to an improved bone health, which agrees to the results of the present study. Overall, given the strong evidence of the association between MF and bone health, one of the main aims during childhood and adolescence should be to increase peak bone mass and increase lean mass through activities to develop and improve MF [15].

On the other hand, some evidence suggests that children who are overweight or obese tend to show higher bone parameters than their healthy-weight peers [2], although this improved bone health in obese children seems to be explained by increased lean mass [38]. Nevertheless, it should be taken into account that excessive adipose tissue accumulation has a negative effect on bone health which may be related to adverse metabolic consequences [39]. In fact, bone accrual in children who are overweight or obese is affected by different humoural stimuli such as inflammatory cytokines, myokines and adipokines including leptin [40]. An article by Gil-Cosano et al. [41] showed that leptin levels were negatively correlated with lumbar spine BMC in overweight or obese children, but a high MF at the lower body could counteract this association, as those children whose maximal repetition at leg press test was above 133.3 kg could overcome the negative influence of leptin. This means a positive effect of MF on bone health, in agreement with the outcomes shown in the present study.

In addition, inflammatory mechanisms worsen bone health in children with overweight or obesity, but higher MF (assessed by a combination of the standardized values of 1RM bench press and 1RM leg press tests) may also attenuate the adverse effect of high tumour necrosis factor-α and vascular endothelial growth factor A on bone mass [13]. These results reinforce those of the present article in relation to the positive effects of absolute strength on bone parameters. Children and adolescents with overweight or obesity are often less skilled than their healthy-weight peers [42], so a stronger correlation of absolute strength with bone health rather than weight-bearing strength is an expected result. The paediatric inactivity triad is a concept that involves exercise deficit disorder, paediatric dynapenia and physical illiteracy [12]. This triad reflects a public health crisis in children and adolescents nowadays, which expresses the tendency of children to be weaker, less agile, slower, less active and therefore with more excess of weight. The paediatric inactivity triad evidences the interrelationship between MF and motor skills, although the results of the present study seem to indicate that absolute strength is more related to bone health than dynamic strength, which requires greater motor skill.

## Strengths and limitations

Some limitations must be considered in this study. The number of participants was low to conduct a cluster analysis with four groups. This could make it more difficult to find differences between groups. On the other hand, another important limitation is the cross-sectional design that precludes the examination of variable changes over time, which would be valuable for investigating how improvements in muscular fitness could impact bone health. Moreover, the participants with the highest body fat could not fit on the DXA scanning table or in the PQCT cavity where the calf is placed.

However, some strengths can be highlighted. The main strength lies in the muscular fitness assessment protocol, employing rigorously validated tests and advanced equipment such as force platforms or strain gauges. Furthermore, DXA and PQCT scans of each participant to obtain data on bone health, both total body and body segment bone density and bone mineral content, as well as bone structure, geometry and strength along with density in different cross-sectional segments.

## Conclusions

In conclusion, a high muscle fitness level shows positive effects on bone health in children with overweight and obesity. Those with the highest absolute maximum isometric strength seemed to have an improved bone health compared to the others, specifically, in polar strength strain index, fracture load and cortical bone area. These findings suggest the importance of promoting MF though resistance training for bone health in overweight or obese children.

## Data Availability

The data presented in this study are available on request from the corresponding author.
